# Geriatric assessment domains to predict overall survival in older cancer patients: An analysis of functional status, comorbidities, and nutritional status as prognostic factors

**DOI:** 10.1002/cam4.3205

**Published:** 2020-07-02

**Authors:** Toshitaka Morishima, Akira Sato, Kayo Nakata, Isao Miyashiro

**Affiliations:** ^1^ Cancer Control Center Osaka International Cancer Institute Osaka Japan

**Keywords:** geriatric assessment, medical record linkage, multicenter study, neoplasms, nutritional status, physical functional performance

## Abstract

Cancer treatments for older patients must account for heterogeneity in health and functional status. Guidelines advocate the use of geriatric assessments (GAs), but comprehensive assessments are laborious and the utility of specific GA domains remains unclear. The identification of specific domains as prognostic factors may support survival predictions and treatment decisions. We aimed to evaluate the associations between several GA domains and overall survival in older cancer patients. We linked cancer registry data and administrative claims data from cancer patients residing in Osaka Prefecture, Japan. The subjects were patients aged ≥70 years who received a diagnosis of gastric, colorectal, or lung cancer between 2010 and 2014 at 36 designated cancer care hospitals. The following three GA domains were assessed at cancer diagnosis: functional status through activities of daily living (ADL), comorbidities, and nutritional status through body mass index. Cox proportional hazards models were constructed for the three cancer types to estimate each domain's prognostic effect while adjusting for gender, age, and cancer stage. Adjusted hazard ratios (HRs) for all‐cause mortality were calculated. We identified 5,559, 4,746, and 4,837 patients with gastric, colorectal, and lung cancer respectively. ADL impairment (HRs: 1.39‐3.34, 1.64‐2.86, and 1.24‐3.21 for gastric, colorectal, and lung cancer, respectively), comorbidities (1.32‐1.58, 1.33‐1.97, and 1.19‐1.29 for gastric, colorectal, and lung cancer, respectively), and underweight (1.36, 1.51, and 1.54 for gastric, colorectal, and lung cancer, respectively) were significantly associated with poorer overall survival. In contrast, overweight was significantly associated with improved overall survival (HRs: 0.82 and 0.89 for gastric and lung cancer respectively). The addition of the three domains increased the models’ C‐statistics (0.816 to 0.836, 0.764 to 0.787, and 0.759 to 0.783 for gastric, colorectal, and lung cancer respectively). Incorporating these factors into initial patient evaluations during diagnosis may aid prognostic predictions and treatment strategies in geriatric oncology.

## Graphical abstract

Comprehensive geriatric assessments aid oncologists’ understanding of heterogeneity in older cancer patients’ health statuses, but the associations between specific domains of geriatric assessments and survival are unclear. In this record‐linked database analysis of older patients with (A) gastric, (B) colorectal, and (C) lung cancer, the authors identified impaired functional status, comorbidities, and underweight at cancer diagnosis as negative prognostic factors; overweight was a positive prognostic factor. Assessments of these factors during cancer diagnosis may support prognostic predictions.

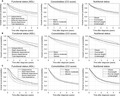

## 1 INTRODUCTION

Rapid demographic transitions and longer life expectancies have contributed to a steady increase in the number of older cancer patients. In 2012, adults aged 65 years and older accounted for 47.5% of all new cancer cases worldwide.^1^ The proportions of older cancer patients will likely continue to rise, particularly in industrialized countries, over the next few decades.^1^

Cancer characteristics and treatment responsiveness tend to differ between younger and older patients. Furthermore, the presence of heterogeneity in aging can complicate the clinical decision‐making process.^2^ The use of chronologic age alone to determine treatment strategies increases the risk of exposing older patients to overtreatment (eg treatment‐related complications) or undertreatment (eg compromised treatment plans).^2^, ^3^ It is therefore important to identify other age‐related prognostic factors (eg frailty and functional reserve) that can support survival predictions and treatment decisions.^4^, ^5^, ^6^, ^7^ Geriatric assessments (GAs) have been advocated to provide better estimations of residual life expectancy and assist treatment decisions in geriatric oncology.^4^, ^5^, ^6^ GAs typically comprise several domains, including functional status, physical performance, fall risk, comorbidities, depressive symptoms, cognition, psychological state, nutritional status, social support, and polypharmacy status.^4^, ^5^, ^6^ The International Society for Geriatric Oncology, National Comprehensive Cancer Network, and American Society of Clinical Oncology recommend that GAs should be performed in all older cancer patients who will receive cancer therapy (irrespective of modality)^4^, ^5^ or all older cancer patients who will receive chemotherapy^6^ to identify vulnerabilities that are potentially overlooked in routine care. While there are numerous assessment tools for each GA domain, the choice of tool varies depending on local preferences, objectives, and available resources.^2^, ^4^, ^5^, ^6^

Studies have reported several GA domains to be independent predictors of patient mortality.^8^, ^9^, ^10^, ^11^, ^12^, ^13^, ^14^, ^15^, ^16^, ^17^ Among these, impaired functional status, comorbidities, and malnutrition are consistently identified as mortality risk factors.^18^, ^19^ Functional status measures in geriatric oncology commonly involve evaluations of activities of daily living (ADL) and instrumental ADL,^4^, ^5^, ^6^ where the former encompasses basic self‐care skills for independent home‐based living and the latter encompasses more complex skills for independent community‐based living. Comorbidities refer to the presence of one or more disorders in addition to the index disease.^20^ These conditions become increasingly prevalent with advancing age,^21^ and are associated with poorer outcomes in older cancer patients.^22^ Malnutrition is a known determinant of mortality in both younger and older cancer patients.^23^ In contrast, younger cancer patients who are overweight or obese have exhibited lower mortality rates than normal‐weight patients.^24^ However, little is known about the association of overweight and obesity with overall survival in older cancer patients.^25^, ^26^

Despite increasing knowledge on the impact of various GA domains on overall survival in cancer patients, prior studies have generally used study populations involving mixed cancer types,^8^, ^9^, ^10^, ^11^ small sample sizes,^8^, ^9^, ^10^, ^13^, ^14^ or single institutions.^9^, ^10^ In order to more accurately identify prognostic factors from GA domains that would inform survival predictions and treatment decisions in older cancer patients, large‐scale studies that examine the relationship between individual domains and survival for specific cancer types should be conducted.^18^, ^19^ In this multicenter retrospective study, we aimed to evaluate the cancer type‐specific associations between several GA domains and overall survival in older patients with gastric, colorectal, and lung cancer.

## 2 METHODS

### 2.1 Data sources

This study linked two data sources to produce a large database that enabled analyses of the associations between clinical information and mortality.^27^, ^28^ The first data source was the Osaka Cancer Registry (OCR), which collects and aggregates population‐based information on cancer diagnoses and outcomes in residents of Osaka Prefecture, Japan. The OCR includes data on patient gender, age at cancer diagnosis, vital status, date of death or the last follow‐up for vital status, date of cancer diagnosis, cancer type, tumor histology, and Surveillance, Epidemiology, and End Results summary stage (ie localized, regional, and distant) at diagnosis.^29^

The second data source was administrative data produced under Japan's Diagnosis Procedure Combination (DPC) Per‐Diem Payment System, which determines insurer reimbursements to acute care hospitals for the provision of healthcare goods and services. In addition to insurance claims, DPC data include clinical summaries for each patient. We collected DPC data from 36 designated cancer care hospitals (designated as such by the national or prefectural government) in collaboration with the Council for Coordination of Designated Cancer Care Hospitals in Osaka.

Patients were linked across both data sources using their hospital‐assigned identification numbers as linkage keys.^30^ The resulting record‐linked database contained approximately half of all new cancer cases in Osaka Prefecture during the study period. Approximately 98% of eligible cancer patients in the OCR database were matched with their corresponding DPC data.

### 2.2 Study population

The target cancer types (gastric, colorectal, and lung) were identified using the topography codes of the International Classification of Diseases for Oncology, Third Edition (ICD‐O‐3). We first identified patients aged 70 years or older (n = 15,994) who received a diagnosis of gastric (C16.x), colorectal (C18.x, C19.x, C20.x), or lung (C33.x, C34.x) cancer between January 1, 2010 and December 31, 2014, and had been admitted to any of the 36 target hospitals during the period spanning 3 months before to 3 months after the index cancer diagnosis. These three cancer types were selected because they are the most commonly occurring cancers in Osaka Prefecture, and accounted for 44% of all new cancer cases in 2014.^31^ We used an age cut‐off of 70 years because of its frequent use in geriatric oncology studies as the age for implementing GA.^4^, ^18^ Patients were excluded if they had a diagnosis of carcinoma *in situ* (n = 666), sarcoma (ICD‐O‐3 morphology codes: 8800‐8921, 8936, 8990‐8991, 9040‐9044, 9120‐9133, 9150, and 9540‐9581; n = 62), lymphoma (ICD‐O‐3 morphology codes: 9590‐9699; n = 13), or missing vital status (n = 111). Patients were monitored until May 2018 using resident registries to verify their vital statuses.

### 2.3 Geriatric assessment domains

The following three GA domains were assessed: functional status, comorbidities, and nutritional status. These were identified at the time of admission based on the relevant data fields in the first inpatient DPC data file during each patient's study period (3 months before to 3 months after cancer diagnosis). This relatively long study period provided a substantial duration to include the index cancer diagnosis and its associated assessments.

Functional status was assessed through the Barthel Index score,^32^ which uses 10 items to measure performance in ADL. These items address continence and independence in bathing, feeding, dressing, using the bathroom, getting up, and moving around the house. A total score ranging from 0 to 100 was calculated for each patient, with higher scores indicating greater levels of independence. Based on a previous study, the following six categories of functional status were used: independence (Barthel Index score: 100), slight dependence (91‐99), moderate dependence (61‐90), severe dependence (21‐60), total dependence (0‐20), and unknown.^32^

Comorbidities were assessed using the updated Charlson Comorbidity Index (CCI) score, which includes 11 comorbid conditions (congestive heart failure, dementia, chronic pulmonary disease, connective tissue disease, mild liver disease, moderate or severe liver disease, diabetes with chronic complications, hemiplegia or paraplegia, renal disease, hematological and solid cancer diagnosed before the cancer of interest, and human immunodeficiency virus infection) identified in 2011 by Quan et al^33^ Comorbidities are recorded in separate DPC data fields using International Classification of Diseases, Tenth Edition codes, and the presence of each comorbidity was identified using an algorithm developed for these codes.^34^ Each comorbid condition was assigned a weight (ranging from 1 to 4) based on its mortality risk^33^; these weights were subsequently summed to calculate each patient's CCI score. Metastatic cancer was excluded from the CCI for this study due to its possible association with the cancer of interest. The following three categories of comorbidities were used: none (CCI score: 0), mild‐to‐moderate (1‐2), and severe (≥3).

Body mass index (BMI) at cancer diagnosis was used as a proxy of nutritional status due to its frequent use in GAs.^4^, ^18^ Although nutritional assessment tools (such as the Mini Nutritional Assessment) may be more accurate measures of nutritional status, we aimed to focus on a simple, easy‐to‐use measure that is routinely used in the clinical setting. BMI was calculated as weight (kg) divided by height (m^2^), and classified into the following five categories used by the World Health Organization: underweight (BMI: <18.5), normal weight (18.5‐24.9), overweight (25.0‐29.9), obese (≥30), and unknown.^35^

### 2.4 Statistical analyses

Categorical variables were calculated as numbers and percentages, and Pearson's chi‐square test was used to compare the results among the three cancer types. Continuous variables were calculated as medians with interquartile range, and the Kruskal‐Wallis test was used to compare the results among the three cancer types. The Kruskal‐Wallis test was chosen as the follow‐up duration had a skewed distribution. To examine the correlations among the three GA domains, Pearson's correlation coefficients were estimated with each domain as a continuous variable; the “unknown” category of each domain was excluded.

A Cox proportional hazards model was constructed for each cancer type to estimate the independent effects of the three GA domains on overall survival for a maximum of five years of follow‐up after adjusting for gender, age (70‐74, 75‐79, 80‐84, and ≥ 85 years), and cancer stage. Tumor histology (small cell carcinoma indicated by ICD‐O‐3 morphology codes: 8041‐8045; nonsmall cell carcinoma indicated by all other ICD‐O‐3 morphology codes) was also included as a covariate for lung cancer patients as it is a known prognostic factor.^36^ The primary outcome was overall survival time, which was defined as the time duration from the date of cancer diagnosis to the date of death from any cause or the date at which a patient was last known to be alive. The assumption of proportional hazards was confirmed through visual inspection of the log‐log survival curve plots (data not shown). The estimated effects of the three GA domains on overall survival were calculated as hazard ratios (HRs) and 95% confidence intervals (CIs). Adjusted survival curves stratified by the categories (excluding the “unknown” category) of the three GA domains were also generated for each cancer type.^37^ To examine the effects of the three GA domains on overall survival according to cancer stage, we constructed a Cox proportional hazards model for each cancer type stratified by stage (excluding the “unknown” category) while adjusting for gender, age, and tumor histology (for the lung cancer model only).

To assess the incremental prognostic value of each GA domain, we estimated Harrell's concordance statistic (C‐statistic) of the different models for each cancer type after excluding patients with an “unknown” cancer stage or domain category.^38^ The C‐statistic is equivalent to the area under the receiver operating characteristic curve, where a value of 0.5 indicates random predictions and a value of 1.0 indicates perfect discrimination between survivors and nonsurvivors. The first model was a “basic” model that controlled for gender, age, cancer stage, and tumor histology (for the lung cancer model only). The GA domains were then added one by one to the basic model. The final model was a “full” model that, in addition to the covariates in the basic model, also included all three GA domains.

The Cox proportional hazards analyses and adjusted survival curve analyses were performed using SAS version 9.4 (SAS Institute). All other analyses were performed using IBM SPSS Statistics version 22 (SPSS Inc). Two‐sided *P* values below .05 were considered statistically significant. The study was approved by our institutional review board.

## 3 RESULTS

### 3.1 Patient characteristics

**FIGURE 1:**
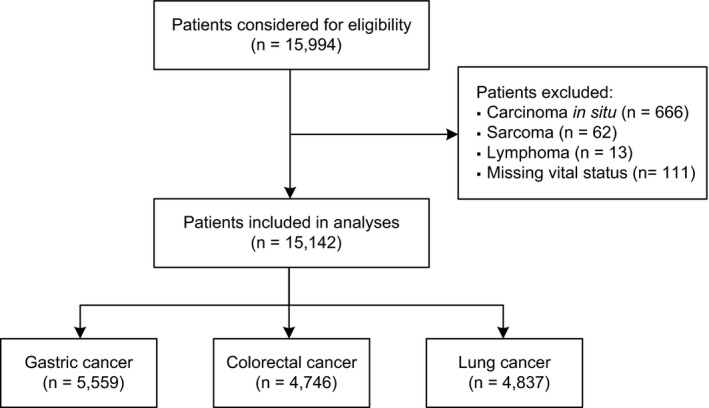
Study flow diagram

A flow diagram of the patient selection process is presented in Figure 1. From 15 994 patients considered for eligibility, we identified 15 142 patients from 35 hospitals as study subjects (one target hospital had no eligible patients). The patient characteristics are summarized in Table 1. The analysis was conducted using 5559 gastric cancer patients, 4746 colorectal cancer patients, and 4837 lung cancer patients. During the 5‐year follow‐up period, the all‐cause mortality rates were 41.7%, 38.1%, and 69.1% in gastric, colorectal, and lung cancer patients respectively. The median follow‐up periods were 3.83, 3.95, and 1.70 years for gastric, colorectal, and lung cancer patients respectively. There were higher proportions of men than women for all cancer types. Approximately 40% of the patients were aged 70‐74 years at cancer diagnosis, and 10% were aged ≥85 years. The most common cancer stage was “localized” in gastric and colorectal cancer patients, and “distant” in lung cancer patients. We observed significant differences among the cancer types in all‐cause mortality, follow‐up duration, and distributions of gender, age, and cancer stage.

**TABLE 1:** Patient characteristics and geriatric assessment domains according to cancer type

	Gastric cancer	Colorectal cancer	Lung cancer	*P* value
Total number of patients	5559 (100)	4746 (100)	4837 (100)	
All‐cause mortality	2316 (41.7)	1806 (38.1)	3342 (69.1)	<.001
Median follow‐up duration, years [IQR]	3.83 [1.55‐4.78]	3.95 [2.48‐5.00]	1.70 [0.62‐3.96]	<.001
Gender				<.001
Female	1620 (29.1)	2109 (44.4)	1562 (32.3)	
Age				<.001
70‐74 y	2092 (37.6)	1725 (36.3)	1945 (40.2)	
75‐79 y	1829 (32.9)	1497 (31.5)	1622 (33.5)	
80‐84 y	1095 (19.7)	902 (19.0)	924 (19.1)	
≥85 y	543 (9.8)	622 (13.1)	346 (7.2)	
Cancer stage at diagnosis				<.001
Localized	3298 (59.3)	2317 (48.8)	1591 (32.9)	
Regional to lymph nodes	633 (11.4)	954 (20.1)	595 (12.3)	
Regional by direct extension	485 (8.7)	580 (12.2)	583 (12.1)	
Distant	1061 (19.1)	796 (16.8)	1942 (40.1)	
Unknown	82 (1.5)	99 (2.1)	126 (2.6)	
Tumor histology				—
Small cell carcinoma	—	—	586 (12.1)	
Functional status (ADL)				<.001
Independence	4584 (82.5)	3667 (77.3)	3738 (77.3)	
Slight dependence	130 (2.3)	100 (2.1)	85 (1.8)	
Moderate dependence	378 (6.8)	371 (7.8)	416 (8.6)	
Severe dependence	267 (4.8)	330 (7.0)	295 (6.1)	
Total dependence	179 (3.2)	257 (5.4)	280 (5.8)	
Unknown	21 (0.4)	21 (0.4)	23 (0.5)	
Comorbidities (CCI score)				<.001
None (0)	4196 (75.5)	3488 (73.5)	3297 (68.2)	
Mild‐to‐moderate (1‐2)	1185 (21.3)	1085 (22.9)	1331 (27.5)	
Severe (≥3)	178 (3.2)	173 (3.6)	209 (4.3)	
Nutritional status				.009
Normal weight	3651 (65.7)	3027 (63.8)	3090 (63.9)	
Underweight	720 (13.0)	669 (14.1)	677 (14.0)	
Overweight	942 (16.9)	801 (16.9)	803 (16.6)	
Obese	87 (1.6)	113 (2.4)	91 (1.9)	
Unknown	159 (2.9)	136 (2.9)	176 (3.6)	

Note

Values are expressed as number (column percentage) unless otherwise indicated. Percentages may not add up to 100% because of rounding.

Abbreviations: ADL, activities of daily living; CCI, Charlson Comorbidity Index; IQR, interquartile range.

Table 1 also shows the distribution of variables in the three GA domains according to cancer type. For functional status, 82.5%, 77.3%, and 77.3% of patients were independent; 2.3%, 2.1%, and 1.8% had slight dependence; 6.8%, 7.8%, and 8.6% had moderate dependence; 4.8%, 7.0%, and 6.1% had severe dependence; and 3.2%, 5.4%, and 5.8% had total dependence for gastric, colorectal, and lung cancer respectively. For comorbidities, 75.5%, 73.5%, and 68.2% of patients had no comorbidities; 21.3%, 22.9%, and 27.5% had mild‐to‐moderate comorbidities; and 3.2%, 3.6%, and 4.3% had severe comorbidities for gastric, colorectal, and lung cancer respectively. For nutritional status, 65.7%, 63.8%, and 63.9% of patients were in the normal weight category; 13.0%, 14.1%, and 14.0% were in the underweight category; 16.9%, 16.9%, and 16.6% were in the overweight category; and 1.6%, 2.4%, and 1.9% were in the obese category for gastric, colorectal, and lung cancer respectively. The Pearson's chi‐square test showed significant differences in the distributions of the three GA domain variables among the cancer types.

Table A1 (Appendix) shows the Pearson's correlation coefficients among the three GA domains (functional status with comorbidities, functional status with nutritional status, and comorbidities with nutritional status) according to cancer type. For all cancer types, significant correlations were found for combinations of functional status with comorbidities (coefficients: −0.06, −0.11, and −0.06 for gastric, colorectal, and lung cancer, respectively) and functional status with nutritional status (coefficients: 0.15, 0.10, and 0.11 for gastric, colorectal, and lung cancer respectively).

### 3.2 Prognostic value of geriatric assessment domains for overall survival

**FIGURE 2:**
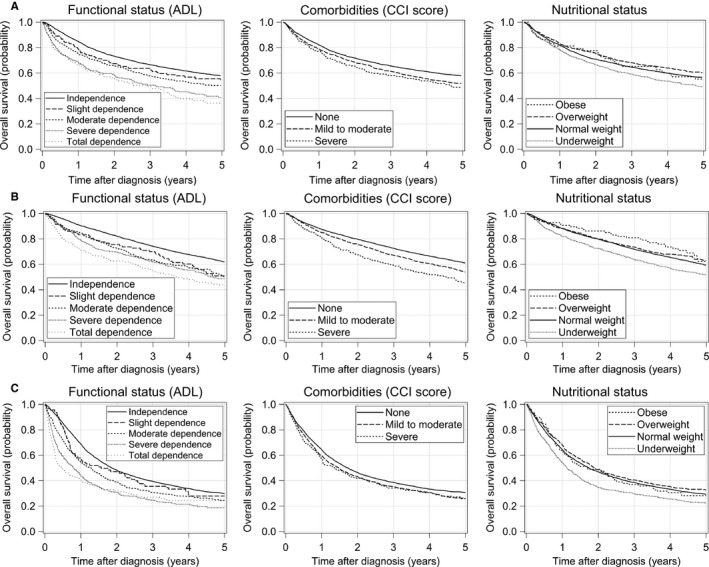
Adjusted overall survival curves for functional status, CCI score, and nutritional status in (A) gastric cancer, (B) colorectal cancer, and (C) lung cancer patients. Gender, age, and cancer stage were adjusted for all survival curves. Tumor histology was also adjusted in lung cancer, but not in gastric cancer and colorectal cancer. Comorbidities and nutritional status, functional status and nutritional status, and functional status and comorbidities were also adjusted in the survival curves for functional status, comorbidities, and nutritional status respectively. ADL, activities of daily living; CCI, Charlson Comorbidity Index

We constructed three separate multivariable Cox proportional hazards models, one for each cancer type. The results for the three GA domains are presented in Table 2, and the results for the baseline patient characteristics (ie gender, age, cancer stage and tumor histology) are presented in Table A2 (Appendix). Figure 2 shows the overall survival curves for each GA domain after adjusting for gender, age, stage, and tumor histology (for lung cancer model only). For all three cancer types, we observed a significant relationship between ADL impairment and all‐cause mortality (Table 2). Gastric cancer patients with slight dependence had a significantly higher hazard of all‐cause mortality (adjusted HR: 1.39; 95% CI: 1.09‐1.77) than functionally independent patients. Moderate dependence had an adjusted HR of 1.68 (95% CI: 1.46‐1.93), severe dependence had an adjusted HR of 2.87 (95% CI: 2.47‐3.34), and total dependence had an adjusted HR of 3.34 (95% CI: 2.81‐3.97) relative to independence. The lung cancer model yielded similar results to the gastric cancer model after also adjusting for tumor histology. In contrast, the colorectal cancer model produced smaller differences in HRs among the different degrees of ADL impairment. Colorectal cancer patients with slight dependence had a significantly higher hazard of all‐cause mortality (adjusted HR: 1.64; 95% CI: 1.24‐2.17) than functionally independent patients. Moderate dependence had an adjusted HR of 1.69 (95% CI: 1.44‐1.97), severe dependence had an adjusted HR of 1.95 (95% CI: 1.67‐2.27), and total dependence had an adjusted HR of 2.86 (95% CI: 2.43‐3.36) relative to independence.

**TABLE 2:** Adjusted hazard ratios and 95% confidence intervals for all‐cause mortality according to cancer type

	Gastric cancer (n = 5,559)			Colorectal cancer (n = 4,746)			Lung cancer (n = 4,837)		
	Mortality (%)	Adjusted HR (95% CI)	*P* value	Mortality (%)	Adjusted HR (95% CI)	*P* value	Mortality (%)	Adjusted HR (95% CI)	*P* value
Functional status (ADL)									
Independence	1610 (35.1)	Reference		1150 (31.4)	Reference		2396 (64.1)	Reference	
Slight dependence	69 (53.1)	1.39 (1.09‐1.77)	.008	52 (52.0)	1.64 (1.24‐2.17)	<.001	63 (74.1)	1.24 (0.96‐1.59)	.094
Moderate dependence	242 (64.0)	1.68 (1.46‐1.93)	<.001	195 (52.6)	1.69 (1.44‐1.97)	<.001	332 (79.8)	1.54 (1.37‐1.73)	<.001
Severe dependence	217 (81.3)	2.87 (2.47‐3.34)	<.001	202 (61.2)	1.95 (1.67‐2.27)	<.001	276 (93.6)	2.48 (2.19‐2.83)	<.001
Total dependence	162 (90.5)	3.34 (2.81‐3.97)	<.001	195 (75.9)	2.86 (2.43‐3.36)	<.001	254 (90.7)	3.21 (2.80‐3.68)	<.001
Unknown	16 (76.2)	3.94 (2.40‐6.49)	<.001	12 (57.1)	1.89 (1.06‐3.36)	.031	21 (91.3)	2.20 (1.40‐3.44)	<.001
Comorbidities (CCI score)									
None (0)	1637 (39.0)	Reference		1214 (34.8)	Reference		2227 (67.5)	Reference	
Mild‐to‐moderate (1‐2)	579 (48.9)	1.32 (1.20‐1.45)	<.001	496 (45.7)	1.33 (1.20‐1.48)	<.001	963 (72.4)	1.19 (1.10‐1.28)	<.001
Severe (≥3)	100 (56.2)	1.58 (1.29‐1.94)	<.001	96 (55.5)	1.97 (1.59‐2.43)	<.001	152 (72.7)	1.29 (1.09‐1.52)	.003
Nutritional status									
Normal weight	1443 (39.5)	Reference		1110 (36.7)	Reference		2084 (67.4)	Reference	
Underweight	455 (63.2)	1.36 (1.22‐1.51)	<.001	343 (51.3)	1.51 (1.34‐1.71)	<.001	558 (82.4)	1.54 (1.40‐1.69)	<.001
Overweight	278 (29.5)	0.82 (0.72‐0.93)	.002	233 (29.1)	0.90 (0.78‐1.04)	.162	488 (60.8)	0.89 (0.81‐0.98)	.023
Obese	28 (32.2)	1.03 (0.71‐1.51)	.86	30 (26.5)	0.74 (0.51‐1.06)	.105	60 (65.9)	1.05 (0.81‐1.36)	.72
Unknown	112 (70.4)	1.58 (1.30‐1.93)	<.001	90 (66.2)	1.55 (1.24‐1.94)	<.001	152 (86.4)	1.87 (1.57‐2.23)	<.001

Note

Mortality is expressed as the number of patients (percentage of all patients at risk as indicated in Table 1). HRs were calculated using Cox proportional hazards models that adjusted for sex, age, and cancer stage. Tumor histology was also adjusted in lung cancer, but not in gastric cancer and colorectal cancer.

Abbreviations: ADL, activities of daily living; CCI, Charlson Comorbidity Index; CI, confidence interval; HR, hazard ratio.

Among gastric cancer patients, all‐cause mortality was significantly higher for patients with mild‐to‐moderate comorbidities (adjusted HR: 1.32; 95% CI: 1.20‐1.45) and severe comorbidities (adjusted HR: 1.58; 95% CI: 1.29‐1.94) when compared with patients with no comorbidities. The colorectal cancer model yielded similar results to the gastric cancer model. In contrast, the lung cancer model yielded smaller differences in HRs among the different degrees of comorbidities. Among lung cancer patients, all‐cause mortality was significantly higher in patients with mild‐to‐moderate comorbidities (adjusted HR: 1.19; 95% CI: 1.10‐1.28) and severe comorbidities (adjusted HR: 1.29; 95% CI: 1.09‐1.52) when compared with patients with no comorbidities.

When compared with normal weight, underweight was significantly associated with higher all‐cause mortality in gastric cancer patients (adjusted HR: 1.36; 95% CI: 1.22‐1.51), colorectal cancer patients (adjusted HR: 1.51; 95% CI: 1.34‐1.71), and lung cancer patients (adjusted HR: 1.54; 95% CI: 1.40‐1.69). However, overweight was significantly associated with lower all‐cause mortality in gastric cancer patients (adjusted HR: 0.82; 95% CI: 0.72‐0.93) and lung cancer patients (adjusted HR: 0.89; 95% CI: 0.81‐0.98) when compared with normal weight. There was no significant association observed between overweight and mortality in colorectal cancer patients (adjusted HR: 0.90; 95% CI: 0.78‐1.04). Obesity was not significantly associated with all‐cause mortality in all three cancer types.

The results of the analyses stratified by cancer stage are provided in Table A3 (Appendix). The cancer stage stratification did not markedly affect the results, which remained similar to those from the models without stratification. Each of the baseline independent variables (ie gender, age, and tumor histology) also yielded HRs similar to those from the models without stratification (data not shown).

### 3.3 Model performance

To estimate the incremental prognostic value of the three GA domains in our statistical models, we compared the C‐statistics of the different models for each cancer type after excluding patients with an “unknown” cancer stage or domain category (Table 3). Among the basic models that contained gender, age, cancer stage, and tumor histology (for the lung cancer model only) as the baseline variables, the models that also contained functional status had higher C‐statistics than those without functional status. The full model with all three GA domains yielded the highest C‐statistics for all three cancer types.

**TABLE 3:** Changes in predictive performance among the different all‐cause mortality models

	Harrell's concordance statistic		
	Gastric cancer (n = 5316)	Colorectal cancer (n = 4514)	Lung cancer (n = 4541)
Basic model	0.816	0.764	0.759
Basic model + functional status	0.830	0.776	0.778
Basic model + comorbidities	0.820	0.773	0.761
Basic model + nutritional status	0.821	0.769	0.766
Basic model + functional status + comorbidities	0.833	0.784	0.779
Basic model + functional status + nutritional status	0.833	0.780	0.782
Basic model + comorbidities + nutritional status	0.825	0.777	0.767
Full model	0.836	0.787	0.783

Note

The basic models included the baseline variables of gender, age at diagnosis, and cancer stage at diagnosis. Tumor histology was also adjusted in lung cancer, but not in gastric cancer and colorectal cancer. The full models included functional status, comorbidities, and nutritional status in addition to the baseline variables.

## 4 DISCUSSION

This large multicenter study provides longitudinal evidence that ADL‐based functional status, comorbidities, and BMI‐based nutritional status assessed at cancer diagnosis have prognostic value for older cancer patients. Because chronologic age alone cannot be the sole determinant of treatment strategies for older cancer patients,^2^, ^3^ several guidelines recommend the implementation of comprehensive GAs involving numerous domains.^4^, ^5^, ^6^ However, comprehensive GAs are time consuming and not all domains have been validated in cancer patients.^39^ Our findings support the use of the above GA domains to improve prognostic accuracy and inform treatment decisions for older cancer patients. Furthermore, we found that functional status made the greatest contribution to our prognostic model among the three domains.

Our analysis found that ADL impairment and the presence of comorbidities at the time of cancer diagnosis were associated with poorer overall survival in older patients, which corroborates the findings of previous reviews.^40^, ^41^, ^42^ Although the most widely used functional scores in oncology involve the assessment of performance status (eg Karnofsky Performance Status and Eastern Cooperative Oncology Group Performance Status), these may underestimate the degree of functional impairment, particularly in older patients.^43^ As an alternative, well‐established ADL evaluation tools such as the Barthel Index are routinely included in GAs.^44^ The association between ADL impairment and poorer overall survival can be explained by an increased risk of postoperative complications, reduced treatment feasibility, and increased chemotoxicity.^40^ Next, the association between comorbidities and poorer overall survival may be due to overtreatment or undertreatment, which can reduce a patient's remaining life expectancy.^41^, ^42^ The prognostic impact of comorbidities observed in this study can also be interpreted as having a direct effect on overall survival.^45^ Our findings support the notion that comorbid medical conditions should be an essential component of GAs.^2^ Interestingly, the HRs for the various degrees of ADL impairment and comorbidities differed among cancer types. We posit that this is due to the varying lethality of malignancies for different cancers, as the overall survival of patients with rapidly growing tumors is more likely to depend on their general health status than comorbidities.

Our findings on the association between low BMI at cancer diagnosis and poorer overall survival are concordant with studies that reported on the negative prognostic impact of malnutrition for various malignancies, including the three types of cancer examined in this study.^46^ Impaired nutritional status in cancer patients is frequently the result of reduced dietary intake (starvation), depleted muscle mass (sarcopenia), and tumor effects (cachexia); these factors can contribute to treatment‐related complications or toxicity.^46^ In contrast, overweight patients had better prognoses than normal‐weight patients in our study. A BMI of 25 kg/m^2^ or higher in the general population is associated with an increased risk of death,^47^ and elevated BMI is linked with increased cancer incidence for several common cancer types.^48^ However, there is emerging evidence that suggests a relationship between obesity and lower mortality risk among patients with various cancers.^49^ This phenomenon is known as the “obesity paradox”, and describes a possible protective effect in overweight and mildly obese patients. The association between higher BMI at cancer diagnosis and better prognosis may be indicative of benefits such as greater muscle mass, additional nutritional reserves, and a reduced risk of dose‐limiting toxicity.^49^ While this phenomenon has been mainly observed among younger cancer patients,^24^ studies have also explored its effects in older patients.^25^, ^26^ Our study contributes to the evidence that overweight is a positive prognostic factor for overall survival in older patients with gastric or lung cancer.

### 4.1 Limitations

This study has several limitations. First, we extracted clinical information from an administrative data source, which is unable to provide a complete or detailed information on GA domains as clinical databases. For example, the data did not include the causes of death, which precluded an examination of disease‐specific mortality. We were also unable to examine other GA domains due to the lack of available data. Second, BMI at cancer diagnosis may be an imprecise indicator of nutritional status in the context of clinical oncology.^49^ The relationship between lower BMI and poorer overall survival may also be explained by methodological biases, such as reverse causality and collider bias.^24^ However, there is no evidence in the existing literature that validates the link between cancer progression and malnutrition in older patients.^46^ Third, some selection bias could not be avoided due to the utilization of a designated cancer care hospital cohort. Such hospitals tend to focus on younger patients with earlier‐stage disease and fewer comorbidities. Thus, our study population may not be representative of the general population. Fourth, CCI scores tend to be underestimated in Japanese administrative data despite the high specificity of comorbid conditions.^50^ Future studies are needed to develop an algorithm for identifying comorbidities from administrative data with improved accuracy. Fifth, the models were not validated using an independent sample, and there is therefore a risk of overfitting. Sixth, we were unable to accurately test the significance between the C‐statistics of the different models as current statistical methods are limited. For example, the "compareC" package in R has been reported to severely inflate type I errors.^51^ Seventh, data limitations prevented us from adjusting for cancer treatment when estimating the effects of the three GA domains on overall survival. Our findings may potentially be explained by the domains’ indirect effects (eg increased mortality due to reduced treatment opportunities or increased complications from overly aggressive treatment) and direct effects (eg increased mortality due to noncancer causes) on overall survival.^4^, ^45^

## 5 CONCLUSIONS

Our study provides evidence on the prognostic value of three GA domains in older cancer patients. Assessing these domains at the point of cancer diagnosis may help to identify potentially overlooked vulnerabilities, provide valuable information on a patient's long‐term overall survival, and contribute to the clinical decision‐making process. Although further studies are needed to verify their utility, these GA domains may help to optimize oncology care for the growing number of older patients with cancer.

## CONFLICT OF INTEREST

All authors declare that they have no conflicts of interest.

## AUTHORS' CONTRIBUTIONS

Toshitaka Morishima MD, PhD: conceptualization, design, acquisition and curation of data, formal analysis, interpretation, drafting of the manuscript, and acquisition of funding. Akira Sato MD, MPH, Kayo Nakata MD, PhD: acquisition and curation of data, interpretation, and critical revision of the manuscript. Isao Miyashiro MD, PhD: conceptualization, acquisition and curation of data, interpretation, critical revision of the manuscript, acquisition of funding, project administration, and supervision.

## Supporting information

Supplementary Material

## Data Availability

The data that support the study's findings are available from the corresponding author upon reasonable request.
