# Supplementary material for: Geriatric assessment domains to predict overall survival in older cancer patients: An analysis of functional status, comorbidities, and nutritional status as prognostic factors
Source: Cancer Med. 2020 Jul 2;9(16):5839–50. doi: 10.1002/cam4.3205 (PMC7433808; doi:10.1002/cam4.3205)
Supplement: Supplementary file 1 — Supplementary Material [file CAM4-9-5839-s001.pdf]

Table A1. Pearson's correlation coefficients among functional status, comorbidities, and nutritional status

		Functional status (ADL)	Comorbidities (CCI score)	Nutritional status
Gastric cancer				
Functional status (ADL)	Coefficient	1.00		
	n	5538		
Comorbidities (CCI score)	Coefficient	−0.06**	1.00	
	n	5538	5559	
Nutritional status	Coefficient	0.15**	−0.01	1.00
	n	5385	5400	5400
Colorectal cancer				
Functional status (ADL)	Coefficient	1.00		
	n	4725		
Comorbidities (CCI score)	Coefficient	−0.11**	1.00	
	n	4725	4746	
Nutritional status	Coefficient	0.10**	−0.04	1.00
	n	4597	4610	4610
Lung cancer				
Functional status (ADL)	Coefficient	1.00		
	n	4814		
Comorbidities (CCI score)	Coefficient	−0.06**	1.00	
	n	4814	4837	
Nutritional status	Coefficient	0.11**	−0.02	1.00
	n	4649	4661	4661

* $P<0.05$. ** $P<0.01$.

n indicates the number of patients (excluding those in the "unknown" category) used to calculate the coefficient.

ADL, activities of daily living; CCI, Charlson Comorbidity Index.

Table A2. Adjusted hazard ratios and 95% confidence intervals for all-cause mortality according to cancer type

	Gastric cancer (n = 5,559)			Colorectal cancer (n = 4,746)			Lung cancer (n = 4,837)		
	Mortality (%)	Adjusted HR (95% CI)	*P* value	Mortality (%)	Adjusted HR (95% CI)	*P* value	Mortality (%)	Adjusted HR (95% CI)	*P* value
Sex									
Female	614 (37.9)	Reference		762 (36.1)	Reference		887 (56.8)	Reference	
Male	1702 (43.2)	1.37 (1.25–1.51)	<0.001	1044 (39.6)	1.26 (1.15–1.39)	<0.001	2455 (75.0)	1.76 (1.63–1.90)	<0.001
Age									
70–74 years	719 (34.4)	Reference		485 (28.1)	Reference		1226 (63.0)	Reference	
75–79 years	717 (39.2)	1.28 (1.16–1.42)	<0.001	538 (35.9)	1.39 (1.23–1.58)	<0.001	1103 (68.0)	1.25 (1.15–1.36)	<0.001
80–84 years	539 (49.2)	1.67 (1.49–1.87)	<0.001	411 (45.6)	1.93 (1.69–2.21)	<0.001	712 (77.1)	1.73 (1.57–1.90)	<0.001
≥85 years	341 (62.8)	2.33 (2.03–2.68)	<0.001	372 (59.8)	2.80 (2.42–3.24)	<0.001	301 (87.0)	2.32 (2.03–2.64)	<0.001
Cancer stage									
Localized	640 (19.4)	Reference		506 (21.8)	Reference		528 (33.2)	Reference	
Regional to lymph nodes	266 (42.0)	2.40 (2.08–2.77)	<0.001	293 (30.7)	1.58 (1.37–1.83)	<0.001	429 (72.1)	3.20 (2.81–3.64)	<0.001
Regional by direct extension	345 (71.1)	5.31 (4.65–6.06)	<0.001	265 (45.7)	2.23 (1.92–2.59)	<0.001	455 (78.0)	3.76 (3.31–4.27)	<0.001
Distant	1001 (94.3)	16.27 (14.61–18.12)	<0.001	687 (86.3)	10.73 (9.51–12.11)	<0.001	1817 (93.6)	7.85 (7.08–8.69)	<0.001
Unknown	64 (78.0)	8.66 (6.69–11.23)	<0.001	55 (55.6)	3.62 (2.73–4.81)	<0.001	113 (89.7)	5.46 (4.43–6.72)	<0.001
Tumor histology									
Non-small cell carcinoma	—	—		—	—		2800 (65.9)	Reference	
Small cell carcinoma	—	—		—	—		542 (92.5)	1.36 (1.23–1.49)	<0.001

Mortality is expressed as the number of patients (percentage of all patients at risk as indicated in Table 1). HRs were calculated using Cox proportional hazards models that adjusted for functional status, comorbidities, and nutritional status. Tumor histology was also adjusted in lung cancer, but not in gastric cancer and colorectal cancer.
CI, confidence interval; HR, hazard ratio.

Table A3. Adjusted hazard ratios and 95% confidence intervals for all-cause mortality according to cancer type stratified by cancer stage

	Gastric cancer				Colorectal cancer				Lung cancer			
	n	Mortality (%)	Adjusted HR (95% CI)	*P* value	n	Mortality (%)	Adjusted HR (95% CI)	*P* value	n	Mortality (%)	Adjusted HR (95% CI)	*P* value
Stage, localized	3298	640 (19.4)			2317	506 (21.8)			1591	528 (33.2)		
Functional status, ADL												
Independence	2917	461 (15.8)	Reference		1882	328 (17.4)	Reference		1371	418 (30.5)	Reference	
Slight dependence	60	12 (20.0)	1.01 (0.57–1.79)	0.98	40	14 (35.0)	1.46 (0.85–2.50)	0.173	28	11 (39.3)	0.99 (0.54–1.83)	0.98
Moderate dependence	177	75 (42.4)	2.85 (2.21–3.67)	<0.001	170	53 (31.2)	1.50 (1.11–2.01)	0.008	112	51 (45.5)	1.46 (1.09–1.97)	0.012
Severe dependence	81	46 (56.8)	3.36 (2.46–4.60)	<0.001	131	58 (44.3)	2.42 (1.81–3.24)	<0.001	43	32 (74.4)	3.57 (2.46–5.17)	<0.001
Total dependence	54	42 (77.8)	7.64 (5.43–10.75)	<0.001	85	49 (57.6)	3.14 (2.28–4.32)	<0.001	36	16 (44.4)	2.00 (1.21–3.31)	0.007
Unknown	9	4 (44.4)	2.01 (0.73–5.55)	0.176	9	4 (44.4)	2.27 (0.83–6.22)	0.11	1	0 (0.0)	0.00 (0.00–>1000)	0.95
Comorbidities (CCI score)												
None (0)	2518	414 (16.4)	Reference		1678	301 (17.9)	Reference		1078	318 (29.5)	Reference	
Mild-to-moderate (1–2)	671	180 (26.8)	1.67 (1.40–1.99)	<0.001	549	162 (29.5)	1.59 (1.31–1.92)	<0.001	428	173 (40.4)	1.57 (1.30–1.90)	<0.001
Severe (≥3)	109	46 (42.2)	2.28 (1.67–3.10)	<0.001	90	43 (47.8)	2.42 (1.74–3.39)	<0.001	85	37 (43.5)	1.72 (1.22–2.42)	0.002
Nutritional status												
Normal weight	2209	389 (17.6)	Reference		1478	301 (20.4)	Reference		1041	322 (30.9)	Reference	
Underweight	322	117 (36.3)	2.06 (1.67–2.54)	<0.001	287	102 (35.5)	1.86 (1.48–2.34)	<0.001	170	88 (51.8)	2.26 (1.78–2.87)	<0.001
Overweight	636	90 (14.2)	0.82 (0.65–1.04)	0.097	442	70 (15.8)	0.86 (0.66–1.12)	0.27	315	93 (29.5)	0.87 (0.69–1.10)	0.24
Obese	63	14 (22.2)	1.26 (0.74–2.16)	0.39	58	11 (19.0)	0.84 (0.46–1.53)	0.56	30	8 (26.7)	1.01 (0.50–2.05)	0.98
Unknown	68	30 (44.1)	1.91 (1.27–2.85)	0.002	52	22 (42.3)	1.53 (0.97–2.42)	0.070	35	17 (48.6)	1.70 (1.04–2.78)	0.035
Stage, regional to lymph nodes	633	266 (42.0)			954	293 (30.7)			595	429 (72.1)		
Functional status, ADL												

Independence	528	202 (38.3)	Reference		788	203 (25.8)	Reference		511	358 (70.1)	Reference	
Slight dependence	16	7 (43.8)	0.88 (0.41–1.90)	0.75	16	5 (31.3)	1.24 (0.50–3.06)	0.64	6	5 (83.3)	1.04 (0.42–2.58)	0.93
Moderate dependence	37	19 (51.4)	1.22 (0.75–1.99)	0.42	62	30 (48.4)	1.89 (1.28–2.79)	0.002	37	30 (81.1)	1.58 (1.08–2.32)	0.019
Severe dependence	30	20 (66.7)	1.67 (1.04–2.68)	0.034	57	35 (61.4)	2.68 (1.84–3.88)	<0.001	26	23 (88.5)	1.95 (1.25–3.06)	0.003
Total dependence	18	14 (77.8)	2.73 (1.57–4.76)	<0.001	27	19 (70.4)	3.46 (2.07–5.78)	<0.001	13	11 (84.6)	5.20 (2.82–9.58)	<0.001
Unknown	4	4 (100.0)	10.03 (3.50–28.68)	<0.001	4	1 (25.0)	0.77 (0.11–5.58)	0.79	2	2 (100.0)	25.37 (5.78–111.35)	<0.001
Comorbidities (CCI score)												
None (0)	452	172 (38.1)	Reference		707	186 (26.3)	Reference		389	269 (69.2)	Reference	
Mild-to-moderate (1–2)	159	82 (51.6)	1.54 (1.17–2.01)	0.002	210	93 (44.3)	1.59 (1.23–2.06)	<0.001	181	139 (76.8)	1.27 (1.03–1.58)	0.025
Severe (≥3)	22	12 (54.5)	1.62 (0.89–2.96)	0.117	37	14 (37.8)	1.29 (0.73–2.28)	0.37	25	21 (84.0)	1.39 (0.88–2.19)	0.157
Nutritional status												
Normal weight	412	168 (40.8)	Reference		635	194 (30.6)	Reference		389	277 (71.2)	Reference	
Underweight	75	40 (53.3)	1.38 (0.97–1.96)	0.077	114	43 (37.7)	1.11 (0.79–1.56)	0.56	65	55 (84.6)	1.66 (1.23–2.25)	<0.001
Overweight	121	46 (38.0)	0.99 (0.71–1.38)	0.95	161	41 (25.5)	0.92 (0.66–1.29)	0.63	115	76 (66.1)	0.92 (0.71–1.19)	0.52
Obese	13	5 (38.5)	0.86 (0.35–2.13)	0.74	28	5 (17.9)	0.56 (0.23–1.36)	0.199	12	9 (75.0)	1.29 (0.63–2.62)	0.48
Unknown	12	7 (58.3)	1.96 (0.90–4.24)	0.089	16	10 (62.5)	1.33 (0.69–2.58)	0.40	14	12 (85.7)	1.75 (0.96–3.17)	0.067
Stage, regional by direct extension	485	345 (71.1)			580	265 (45.7)			583	455 (78.0)		
Functional status, ADL												
Independence	364	241 (66.2)	Reference		406	165 (40.6)	Reference		463	344 (74.3)	Reference	
Slight dependence	16	12 (75.0)	1.27 (0.70–2.31)	0.42	14	6 (42.9)	0.98 (0.43–2.24)	0.96	6	6 (100.0)	4.82 (2.10–11.06)	<0.001
Moderate dependence	50	41 (82.0)	1.54 (1.08–2.18)	0.016	52	30 (57.7)	1.47 (0.98–2.21)	0.060	55	46 (83.6)	1.36 (0.99–1.86)	0.058
Severe dependence	36	33 (91.7)	3.03 (2.04–4.50)	<0.001	50	22 (44.0)	1.07 (0.68–1.67)	0.78	26	26 (100.0)	2.18 (1.44–3.30)	<0.001
Total dependence	17	16 (94.1)	3.52 (2.04–6.06)	<0.001	56	41 (73.2)	1.95 (1.33–2.86)	<0.001	27	27 (100.0)	4.29 (2.87–6.42)	<0.001

Unknown	2	2 (100.0)	42.00 (9.95–177.22)	<0.001	2	1 (50.0)	1.10 (0.15–8.03)	0.93	6	6 (100.0)	1.48 (0.58–3.79)	0.41
Comorbidities (CCI score)												
None (0)	368	258 (70.1)	Reference		434	183 (42.2)	Reference		395	307 (77.7)	Reference	
Mild-to-moderate (1–2)	104	77 (74.0)	1.21 (0.93–1.56)	0.150	126	68 (54.0)	1.26 (0.94–1.68)	0.118	170	132 (77.6)	1.11 (0.90–1.37)	0.33
Severe (≥3)	13	10 (76.9)	0.91 (0.47–1.75)	0.77	20	14 (70.0)	1.80 (1.03–3.13)	0.039	18	16 (88.9)	1.12 (0.67–1.88)	0.67
Nutritional status												
Normal weight	325	230 (70.8)	Reference		364	163 (44.8)	Reference		346	260 (75.1)	Reference	
Underweight	81	66 (81.5)	1.29 (0.97–1.73)	0.085	114	59 (51.8)	1.23 (0.91–1.67)	0.180	115	104 (90.4)	1.95 (1.54–2.46)	<0.001
Overweight	61	33 (54.1)	0.59 (0.41–0.85)	0.004	79	32 (40.5)	1.00 (0.68–1.47)	0.99	84	57 (67.9)	0.91 (0.68–1.22)	0.53
Obese	4	4 (100.0)	4.24 (1.52–11.81)	0.006	10	1 (10.0)	0.18 (0.03–1.28)	0.086	18	14 (77.8)	1.03 (0.59–1.78)	0.92
Unknown	14	12 (85.7)	1.37 (0.75–2.52)	0.31	13	10 (76.9)	2.69 (1.35–5.38)	0.005	20	20 (100.0)	2.25 (1.32–3.84)	0.003
Stage, distant	1061	1001 (94.3)			796	687 (86.3)			1942	1817 (93.6)		
Functional status, ADL												
Independence	729	677 (92.9)	Reference		531	436 (82.1)	Reference		1335	1228 (92.0)	Reference	
Slight dependence	36	36 (100.0)	1.51 (1.08–2.12)	0.016	29	26 (89.7)	1.85 (1.23–2.78)	0.003	42	38 (90.5)	1.19 (0.86–1.64)	0.31
Moderate dependence	106	99 (93.4)	1.33 (1.07–1.65)	0.010	76	73 (96.1)	1.65 (1.27–2.14)	<0.001	194	188 (96.9)	1.58 (1.35–1.85)	<0.001
Severe dependence	106	105 (99.1)	3.08 (2.47–3.84)	<0.001	84	79 (94.0)	1.81 (1.41–2.33)	<0.001	182	179 (98.4)	2.60 (2.21–3.06)	<0.001
Total dependence	79	79 (100.0)	2.63 (2.04–3.39)	<0.001	72	69 (95.8)	2.78 (2.13–3.64)	<0.001	176	172 (97.7)	3.14 (2.65–3.72)	<0.001
Unknown	5	5 (100.0)	3.39 (1.39–8.27)	0.007	4	4 (100.0)	2.12 (0.78–5.79)	0.143	13	12 (92.3)	2.28 (1.27–4.09)	0.006
Comorbidities (CCI score)												
None (0)	796	747 (93.8)	Reference		600	509 (84.8)	Reference		1346	1256 (93.3)	Reference	
Mild-to-moderate (1–2)	232	223 (96.1)	1.09 (0.93–1.26)	0.30	176	158 (89.8)	1.16 (0.97–1.40)	0.109	519	487 (93.8)	1.12 (1.01–1.25)	0.034
Severe (≥3)	33	31 (93.9)	1.27 (0.89–1.83)	0.190	20	20 (100.0)	1.83 (1.16–2.89)	0.009	77	74 (96.1)	1.20 (0.94–1.52)	0.137

Nutritional status												
Normal weight	672	635 (94.5)	Reference		503	429 (85.3)	Reference		1240	1161 (93.6)	Reference	
Underweight	218	208 (95.4)	1.06 (0.91–1.25)	0.44	137	127 (92.7)	1.51 (1.23–1.84)	<0.001	306	291 (95.1)	1.28 (1.13–1.46)	<0.001
Overweight	112	103 (92.0)	0.93 (0.75–1.14)	0.48	105	86 (81.9)	0.94 (0.74–1.19)	0.60	276	251 (90.9)	0.89 (0.78–1.02)	0.103
Obese	6	4 (66.7)	0.53 (0.20–1.42)	0.21	12	10 (83.3)	0.99 (0.53–1.85)	0.96	30	28 (93.3)	1.10 (0.75–1.60)	0.64
Unknown	53	51 (96.2)	1.27 (0.95–1.70)	0.110	39	35 (89.7)	1.46 (1.02–2.08)	0.037	90	86 (95.6)	1.82 (1.44–2.29)	<0.001

Mortality is expressed as the number of patients (percentage of all patients at risk as indicated in the leftmost column). HRs were calculated using Cox proportional hazards models that adjusted for sex, age, and cancer stage. Tumor histology was also adjusted in lung cancer, but not in gastric cancer and colorectal cancer.

ADL, activities of daily living; CCI, Charlson Comorbidity Index; CI, confidence interval; HR, hazard ratio.
